# Indentation probe with optical fibre array‐based optical coherence tomography for material deformation

**DOI:** 10.1111/jmi.12994

**Published:** 2021-01-13

**Authors:** Marica Marrese, E.J. Paardekam, Davide Iannuzzi

**Affiliations:** ^1^ Department of Physics and Astronomy and LaserLab Vrije Universiteit Amsterdam The Netherlands

**Keywords:** optical coherence tomography, optical fiber array, indentation, lensed optical fibers

## Abstract

We present a new optomechanical probe for mechanical testing of soft matter. The probe consists of a micromachined cantilever equipped with an indenting sphere, and an array of 16 single‐mode optical fibres, which are connected to an optical coherence tomography (OCT) system that allows subsurface analysis of the sample during the indentation stroke. To test our device and its capability, we performed indentation on a PDMS‐based phantom. Our findings demonstrate that Common Path (CP)‐OCT via lensed optical fibres can be successfully combined with a microindentation sensor to visualise the phantom's deformation profile at different indentation depths and locations in real time.

**Lay Description:**

This work presents a new approach to simultaneously perform micro‐indentation experiments and OCT imaging. An optical fiber array‐based sensor is used to develop a new hybrid tool where micro‐indentation is combined with optical coherence tomography. The sensor is therefore capable of compressing a sample with a small force and simultaneously collecting OCT depth profiles underneath and around the indentation point. This method offers the opportunity to characterize the mechanical properties of soft materials and simultaneously visualize their deformation profile. The ability to integrate OCT imaging with indentation technology is promising for the non‐invasive and precise characterization of different soft materials.

## 1 INTRODUCTION

The human body is continuously subjected to a variety of external forces while performing a multitude of activities. Therefore, the investigation of mechanical properties is crucial in several research fields and at all length scales, from cellular up to tissue and organ scale.^1^, ^2^

The local mechanical properties of soft biological materials are typically assessed via nano and microindentation by means of Atomic Force Microscopy (AFM).^3^, ^4^, ^5^, ^6^, ^7^ Here, a nanometre‐size tip is mounted on the free‐hanging end of a cantilever to indent the sample with a small force. By measuring the deflection of the cantilever, one can then obtain the elasticity of the sample at nano‐ and microscale.

To contribute to this field, in 2010, our group proposed a new approach to precisely and locally map the tissue's viscoelastic properties at microscale by means of ferrule‐top technology. The technique relies on a ferrule‐top probe, which is obtained by assembling a rather macroscopic (compared to AFM) cantilever on a millimetre‐sized glass ferrule. The cantilever is equipped with a sphere at its free‐hanging end, which is brought in contact with the sample's surface to allow the user to apply the mechanical stimulus. An optical fibre is then used to measure the deflection of the cantilever providing the information needed to assess the mechanical properties of the sample, as in AFM nano‐indentation.^9^, ^10^, ^11^, ^12^, ^13^ Ferrule‐top cantilevers are in general easier to use than AFM's, especially when the sample has to be kept in a liquid environment during the measurement (as is often the case with biological samples). This technique has already been used by several research groups to assess the mechanical properties of biomaterials, tissues and cells (see Refs. 14, 15, 16, 17, 18, 19, 20, 21, 22, 23, 24, 25, 26, 27 and references therein).

Although indentation techniques can accurately probe the local mechanical properties of biological tissue, and simultaneously provide subcellular imaging they are less suitable for in‐depth optical imaging of tissues. Microindentation sensors can, in fact, provide data on the bulk mechanical response of the sample (at tissue level), without the possibility to discriminate subsurface deformations due to the application of the external load. A visualisation of the inner structures of the sample (on a microscale) would provide additional information for a better interpretation of the mechanical properties of the indented material.

Triggered by this series of considerations, in 2013 we demonstrated that the ferrule‐top probes used for indentation can be modified to hold an additional optical fibre that, when connected to an optical coherence tomography (OCT) system, enables one to visualise how the subsurface layers of a sample deform under the application of an external load.^28^ In this design, a hollow tube was used as indenting tip to allow the light for OCT imaging to pass through the probe and reach the sample at the point of indentation. This configuration posed severe limitations on the quantitative analysis of the mechanical data since there are no analytical models for such an indenter shape. To overcome those limitations, a few years later, this approach was further improved^29^ by using a half‐ball lens indenter tip to probe the material. The half‐ball lens indenter was also used as a focusing element for the detection of the OCT signal.

Both the designs incorporate only a single OCT‐fibre, which acquires a one‐dimensional OCT depth profile of the sample at the indenter contact point. However, to visualise the sample's deformation during the indentation stroke, one needs to transversally scan the region of interest and reconstruct a cross‐sectional image of the tissue around the indentation site. To this end, in this paper, we propose a new experimental sensor that combines an array of multi‐OCT fibres with microindentation capability. The instrument is based on a miniaturised cantilever probe that compresses the sample with a small force and simultaneously collects OCT depth profiles around the indentation point. The integration of microindentation and OCT allows one to investigate, in principle, the mechanical properties of any material while acquiring 2D‐profiles that show the tissue deformation during a single indentation stroke in real time. Before going into the details, we believe it is important to clarify that the goal of this paper is to test the working principle of the probe and not to validate its performance in identifying specific mechanical properties of the material. The latter would require an experimental effort that goes beyond the resources currently available. It is, however, fair to remind the reader that the use of ferrule‐top technology for material characterisation has already been discussed in numerous other papers.^11^, ^12^, ^13^, ^32^, ^33^, ^34^

## 2 MATERIALS AND METHODS

### 2.1 Ferrule‐top sensor: design and fabrication

**FIGURE 1:**
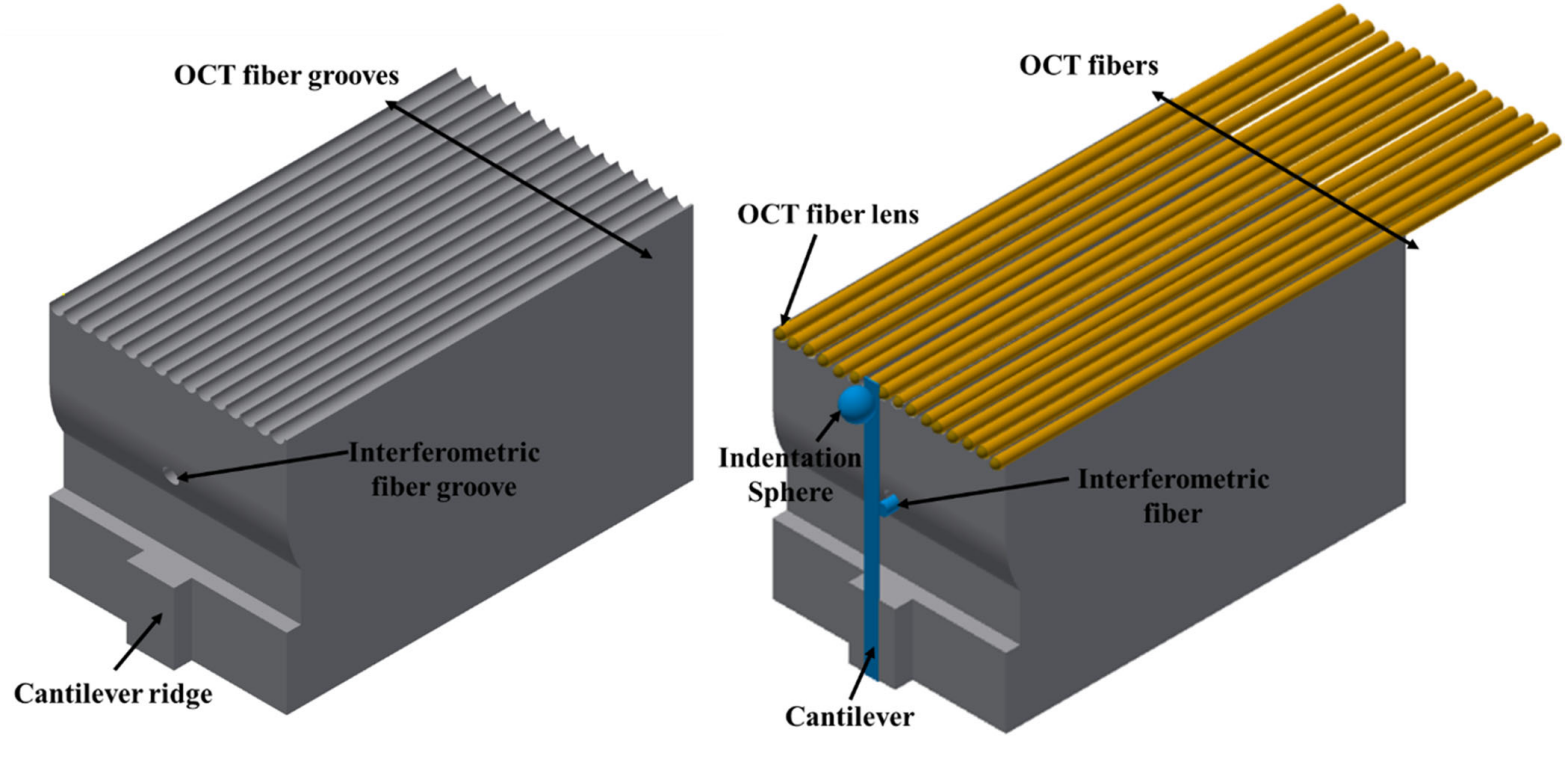
3D printed ferrule‐top probe: (Left) 5 mm × 5 mm × 10 mm 3D printed ferrule. The ridge to mount the cantilever, the interferometric groove, and the 16 OCT fibre grooves are depicted; (Right) front and side view of the ferrule

**FIGURE 2:**
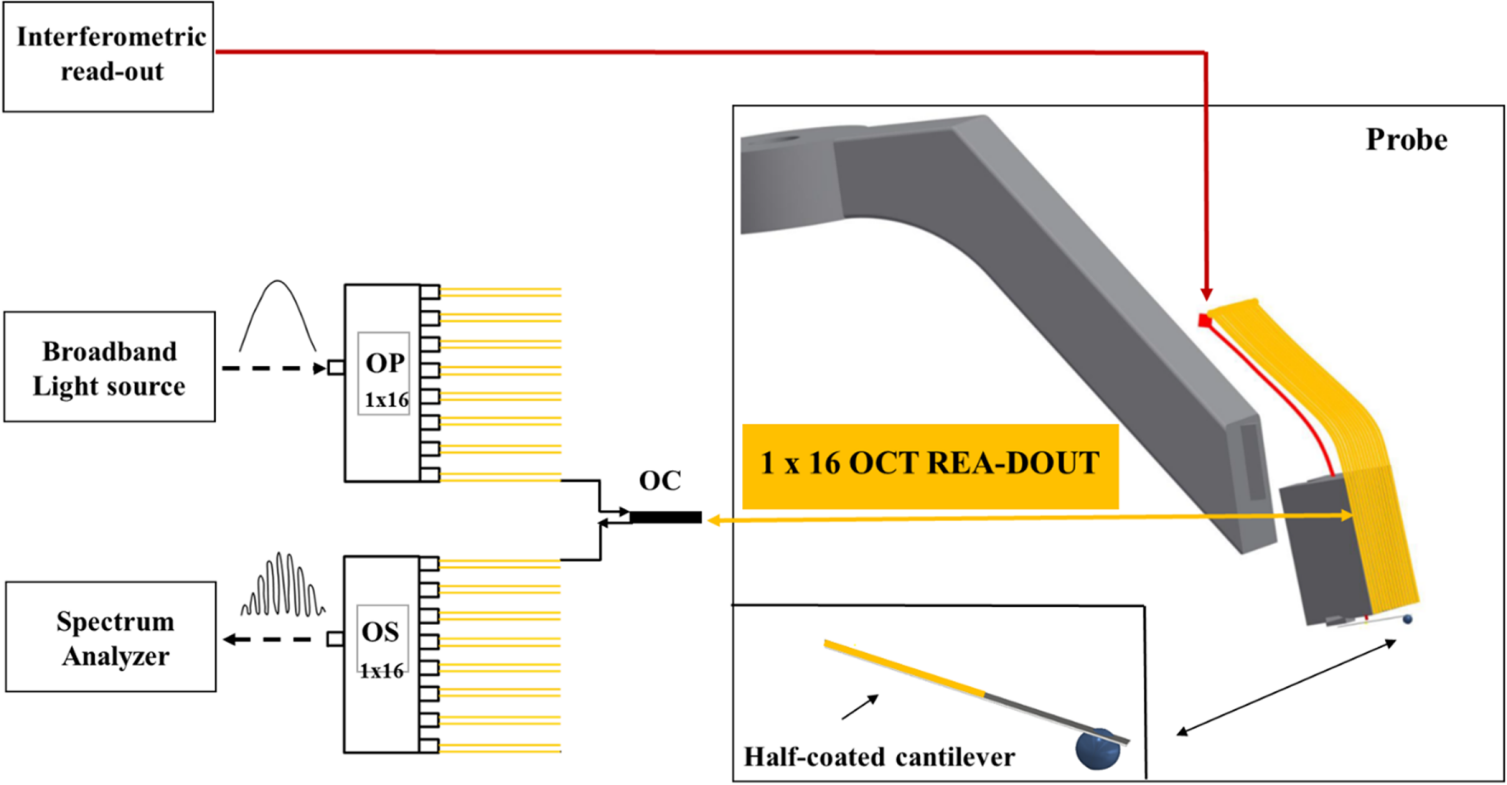
Schematic view of the setup. The OCT fibres (in yellow) are connected to a broadband‐source optical coherence tomography readout via a 1 × 16 optical switch. The back‐reflected light from the sample is coupled back to the detector of the OCT system via an optical coupler. The central optical fibre (in red), connected to an interferometer, allows one to measure the deflection of the cantilever and, therefore, to quantify the mechanical load applied to the sample during indentation. A magnified view of the half‐coated cantilever equipped with a spherical tip employed to probe the sample is shown in the bottom‐right insert

**FIGURE 3:**
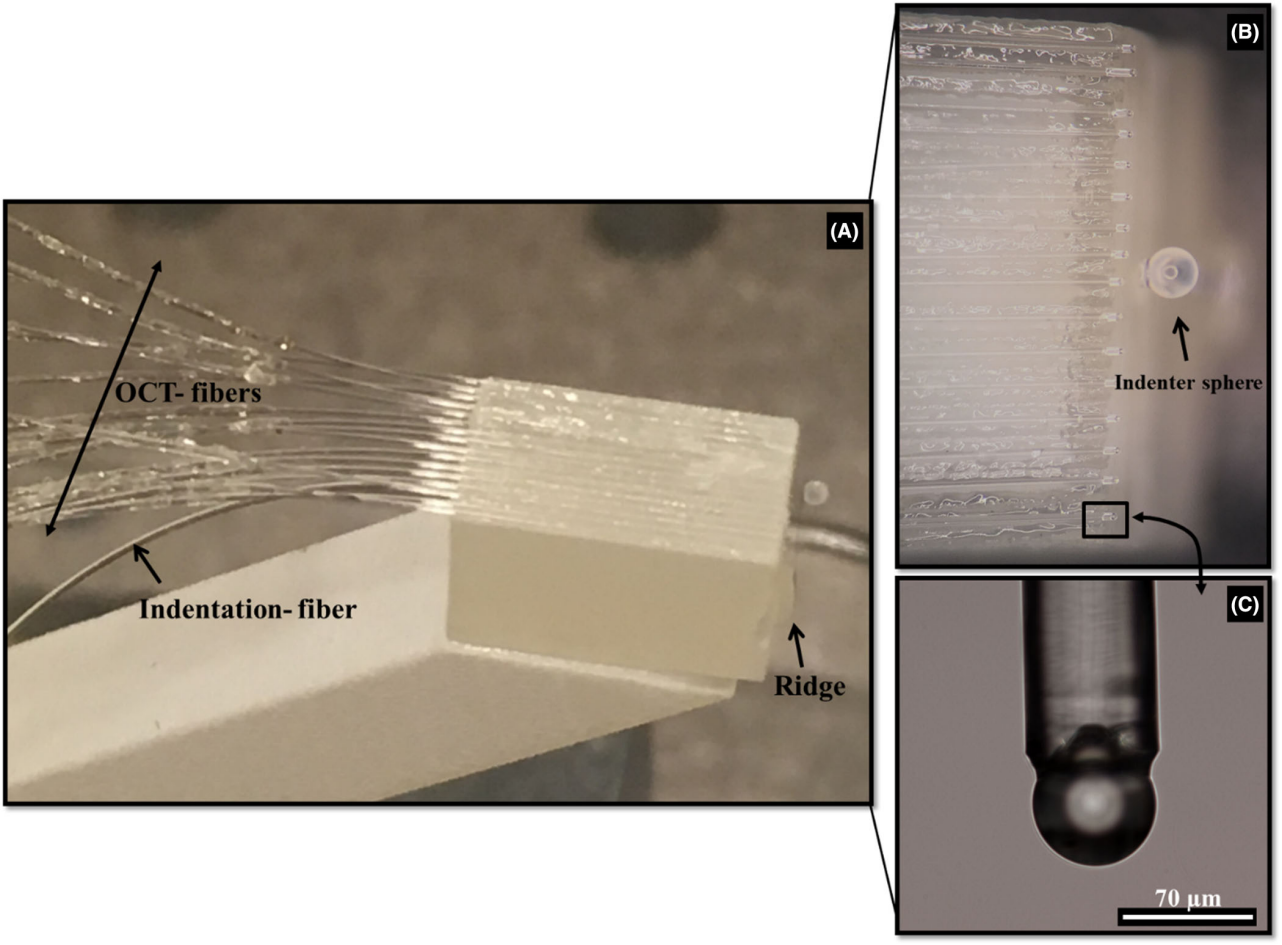
Ferrule‐top sensor: (A) Ferrule‐top sensor combining micro indentation and OCT: 1 × 16 OCT fibres glued on the top‐facet of the 3D ferrule further mounted on a rigid 3D‐printed arm. (B) A magnified optical microscope view of the sensor hosting the OCT fibres. (C) Magnified view of one of the OCT fibre equipped with a spherical lens tip

The building block of the sensor is a 5 mm × 5 mm × 10 mm 3D‐printed ferrule, which has a 300 μm ridge, 1 hole in the centre, and 16 microgrooves on the front side of the rectangular ferrule (pitch ≈ 300 μm), as illustrated in Figure 1. A half gold‐coated borosilicate cantilever (300 µm × 30 µm) is glued on the ridge of the ferrule as in a standard ferrule‐top configuration.^10^, ^30^ The free‐hanging end of the cantilever is equipped with a spherical tip (diameter of 400 µm) to probe the material. Next, a single‐mode optical fibre (corning SMF128) is cleaved and mounted in the central hole of the 3D‐printed ferrule, pointing at the reflective part of the cantilever, and is used to interferometrically read out the deflection of the cantilever during an indentation experiment (Figure 2). The 16 v‐grooves are used to mount 16 single‐mode etched optical fibres, each equipped with *a* ≈ 65 µm diameter barium titanate sphere^31^ (Figure 3).

### 2.2 Experimental setup

The optical fibre mounted in the central groove and aligned with the reflective part of the cantilever is connected to a commercial interferometer (OP1550, Optics11) to measure, via Fabry‐Perot interferometry, the deflection of the cantilever. The 16 OCT fibres are connected to a spectral‐domain OCT system (Telesto II series, Thorlabs GmbH, Germany) implemented in Common Path‐OCT mode (CP‐OCT). The layout of the system is shown in Figure 2. Briefly, the output of a superluminescent diode (SLD, D‐1300 HP, Superlum, Ireland‐with a full‐width half‐maximum of 85 nm and a central wavelength of 1300 nm) is fed to a liquid crystal 1×16 optical switch (CrystaLatchTM 1×16 Fiberoptic Switch, Agiltron, USA) that can reach a nominal switching time of 50 µs. Each fibre of our common path sensor is attached to one of the outputs of the optical switch. The signal collected from each of the fibres is connected to a broadband circulator (CIRC‐3‐31‐PB, Gould Fiber Optics, USA), whose exit is further sent to a 1×16 optical splitter. The output of the optical splitter is then connected to a spectrometer (THORLABS 1310, Wasatch Photonics, Inc.), which consists of an InGaAs line‐scan camera (GL‐2048‐L, Sensors Unlimited, Inc., USA) with 2048 pixels. The raw data are collected and processed using a custom‐designed LabVIEW (National Instruments, USA) interface.

Finally, the sensor is mounted on a piezoelectric stage operating in closed‐loop mode, which is then used to drive the indentation stroke. Briefly, a cantilever with a spring constant in the range of 3.4±0.2 N/m, calibrated according to Ref. (8) and a sphere radius of ∼240 µm was used. Further details of sensor design and the experimental setup that combines optical coherence tomography with depth‐sensing microindentation are reported as supplementary material. The characterisation of the lensed probes has been reported in our previous work.^31^ The initial distance between the probe and sample is determined as a trade‐off between the lens properties (penetration depth of approximately 1.5 mm), the cantilever deflection, and the indentation sphere.

### 2.3 OCT calibration procedure

Before each experiment, two types of calibration are performed: background subtraction and axial offset alignment. The background image is obtained when no scattering material is present in front of the sensor and is then subtracted from all subsequent scans. As far as the axial offset alignment calibration is concerned, one should consider that the facets of the OCT fibres are not perfectly aligned. This misalignment introduces relative axial shifts in the recorded A‐scans. To overcome this issue, the probe is mounted in front of a flat surface, which allows us to measure the phase delay due to the misalignment. The misalignment is then compensated for during the actual indentation measurements. Both calibration procedures are directly implemented in Labview; therefore, no further postprocessing of OCT data is performed. After the OCT calibration, the actual mechanical measurement starts. As the tip is pushed into the sample, the deflection of the cantilever, the indentation depth (obtained subtracting the measured deflection on the cantilever from the vertical motion of the piezoelectric stage to which the probe is anchored), and the 16 OCT depth profiles are simultaneously acquired.

### 2.4 Sample preparation

To test our approach, we used the sensor described above to indent and simultaneously image a PDMS‐based phantom. The sample consists of a ∼300 µm thin membrane, made of Sylgard 184 polydimethylsiloxane (PDMS, 18∶1 elastomer to curing agent ratio, Dow Corning) poured on a plastic ring. To improve the scattering properties of the material, the phantom was prepared with the addition of TiO_2_ particles suspended in OH‐terminated silicone oil, following the fabrication steps indicated in Bartolini et al.^29^

## 3 RESULTS

The indenter is based on ferrule‐top technology,^10^, ^11^, ^30^ where a micromachined cantilever is used to indent the phantom. The OCT fibres detect the sample structure during the indentation stroke, allowing one to visualise how the subsurface layers of the indented material respond to the external load.

Following the production protocol described in the method section, we produced several sensors. For a more systematic use of this probe, it is therefore mandatory to find more suitable (possibly automated) assembly techniques. It is worth stressing that the lensed fibres proved to be prone to breaking during the fabrication phase; furthermore, the (manual) gluing process is rather complex and often leads to nonfully functioning probes. The ultrathin fibres are glued in the u‐groove of a 3D printed fibre array and mounted on a motorised Z‐stage, while the sample is positioned perpendicularly to the fibre axis. However, the resolution of the groove of the 3D printed ferrule does not ensure a straight alignment of the fibres toward the sample, making the coupling back of the light very difficult. This is a severe limitation of this probe. As a matter of fact, the best probe we were able to fabricate could only count 11 working OCT fibres out of the 16 expected.

**FIGURE 4:**
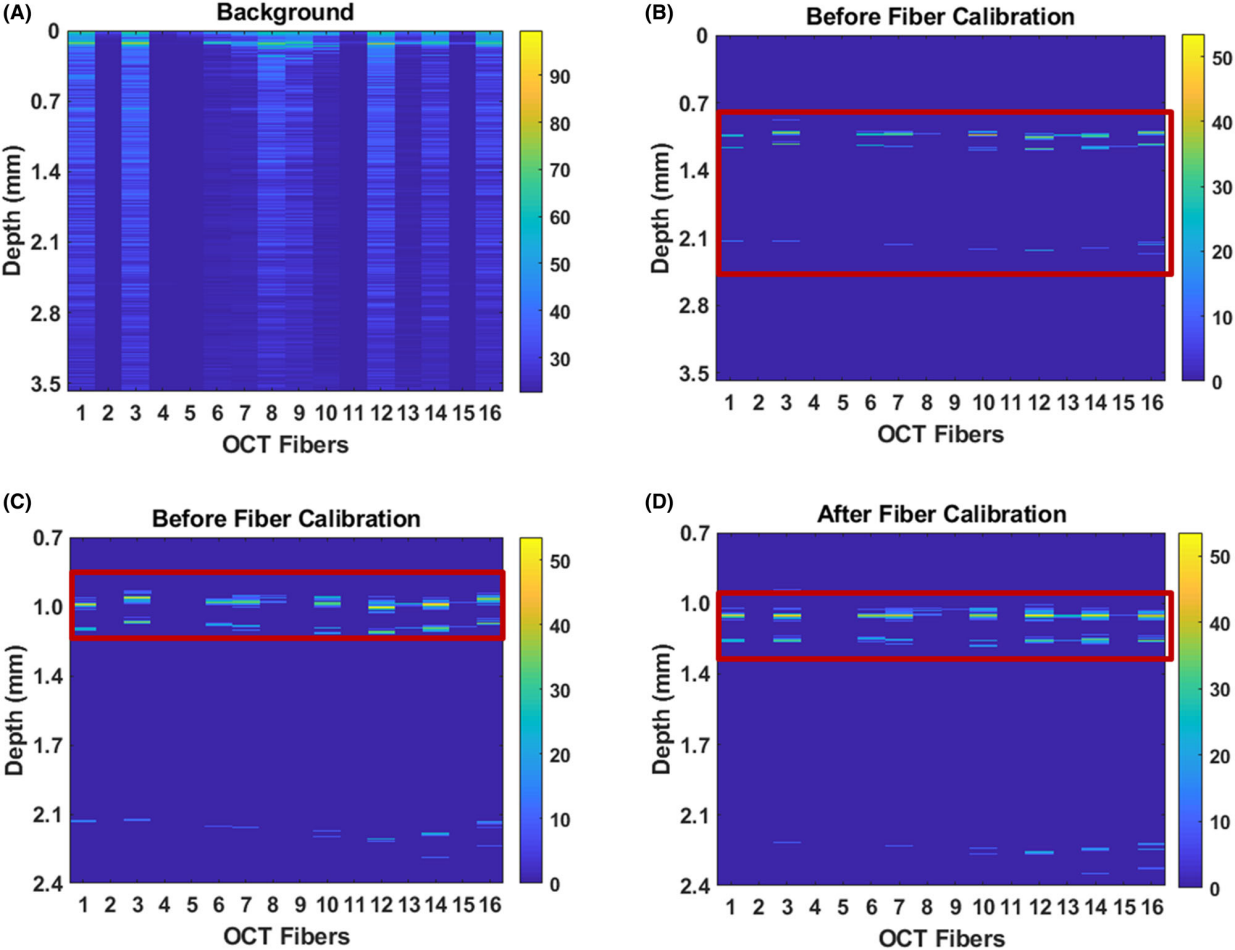
Probe calibrations. Top view: Background OCT scan (A) and OCT image of a flat microscope glass slide (B) before axial shift calibration. Bottom view: zoom‐in image of (C) the microscope glass slide before calibration and (D) B‐scan of the same microscope glass slide after axial shift calibration. The probe is held at ∼1 mm from the glass slide

In our study, we refer to A‐scan as the signal recorded by one single fibre. A set of consecutive A‐scan acquired from several imaging fibres produces a cross‐sectional OCT image called B‐scan. Figure 4 shows the results of the two calibration steps: background subtraction and axial offset alignment. The latter was obtained by juxtaposing the probe and a ∼1 mm thick microscope glass.

As expected, the data acquired before the calibration, reported in Figures 4(B) and (C), result in a nonflat surface due to the misalignment of the OCT fibres in the Z‐direction. On the contrary, in Figure 4(D), the corrected A scan shows that the top and bottom surfaces of the glass microscope slide are well‐aligned. The commercial OCT system used in this study allows one to obtain A‐scans (depth profile) up to 3.5 mm; therefore, to appreciate the misalignment due to the probe fabrication, it is necessary to enlarge the images (Figure 4Bvs 4C – same B‐scan but different *y*‐scale).

**FIGURE 5:**
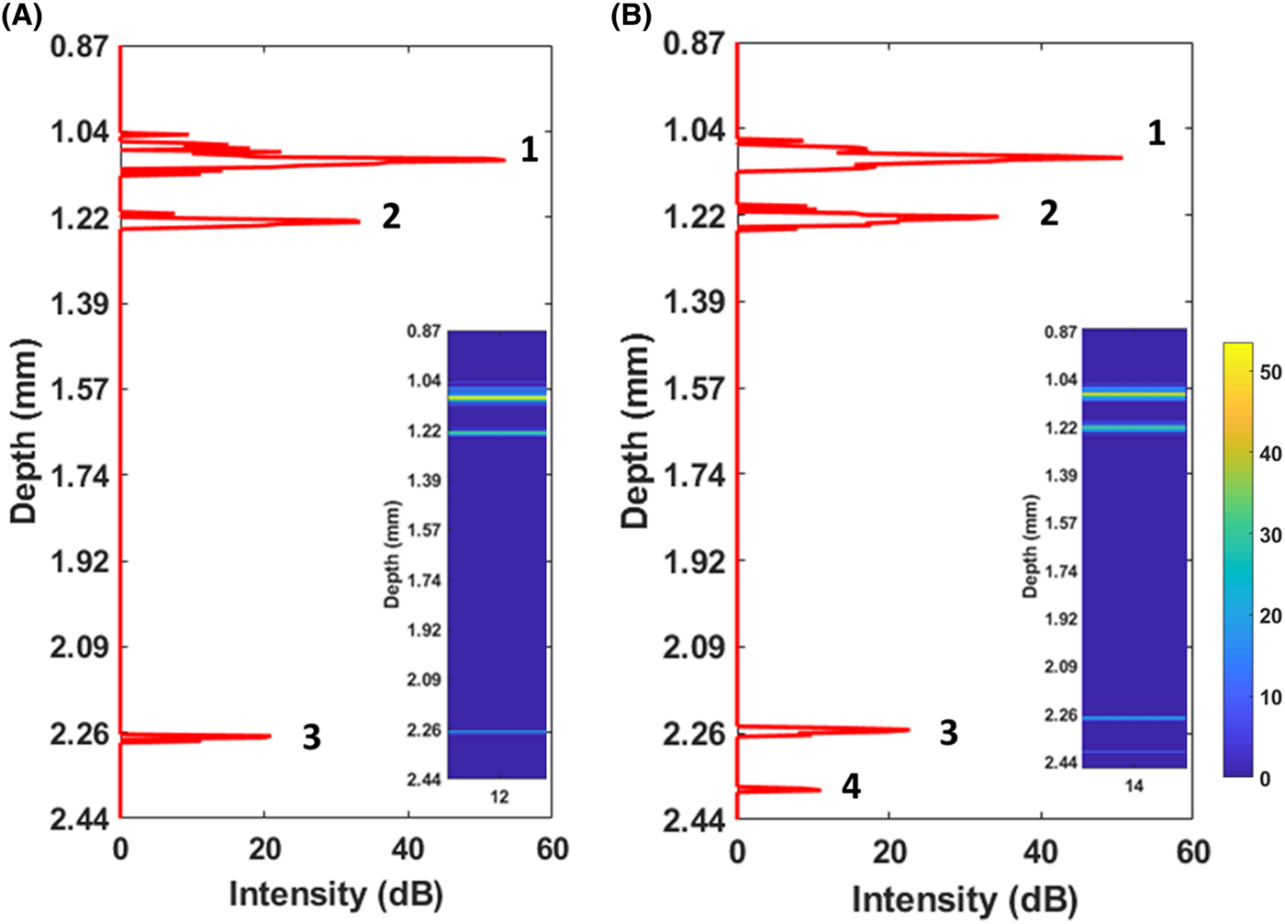
Axial shift calibration. Single OCT A‐scan obtained for (A) fibre 12 and (B) fibre 14 on a microscope glass slide. Peak 1: (fibre‐to‐) lens‐(front facet) glass slide interface; Peak 2: lens (‐to‐air)‐(front facet) glass slide interface; Peak 3: (fibre‐to‐) lens‐glass slide interface and Peak 4: lens (‐to‐air)‐(rear facet) glass slide interface. The probe is held at ∼1 mm from the glass slide. The magnitude scale bar represents the A‐scan intensity in dB

In Figure 5, we further show two typical offset‐corrected A‐scans obtained in this configuration. Each of the A‐scans presents 3 or 4 peaks, depending on the sensitivity of the OCT fibre, and all peaks are well aligned. Specifically, peaks 1 and 2 represent the sphere‐to‐front surface of the microscope slide interface, as first (fibre‐to‐lens) and second reflections (lens‐to‐air), respectively. In contrast, peaks 3 and 4 are the sphere‐to‐rear surface of the glass slide interface, as first and second reflection.

The distances between the small peaks (peak 1 vs peak 2) are ∼125 µm for fibre 12 and ∼118 µm for fibre 14. However, the scale bar refers to the image depth that one would observe in vacuum (*n* = 1.00); therefore, to obtain the correct geometrical distances, one has to consider the effect of the refractive index of the material's light travel through. Considering that the fibre lens is made of barium titanate with a refractive index *n* = 1.95, the peak‐to‐peak geometrical distances (*d_g_* – peak 1 vs peak 2 and peak 3 vs peak 4) correspond indeed to the dimension of the lens (∼ 65 µm in diameter). The effect of the two reference surfaces (front and rear lens facet) adds two additional peaks in the OCT scans. Those kinds of reflections are unavoidable in this type of sensor, as they arise from the reflections due to the several interfaces the light has to encounter before reaching the sample. The *d_g_* for peak 1 to peak 3 and peak 2 to peak 4 has been measured to be ∼0.8 mm. Those distances correspond to the microscope glass slide (typically 1 mm thick). This set of data, together with the graphs showed in Figure 3, also demonstrates that the lens has a penetration depth of at least ∼2.5 mm. The magnitude scale bar represents the A‐scan intensity in dB.

**FIGURE 6:**
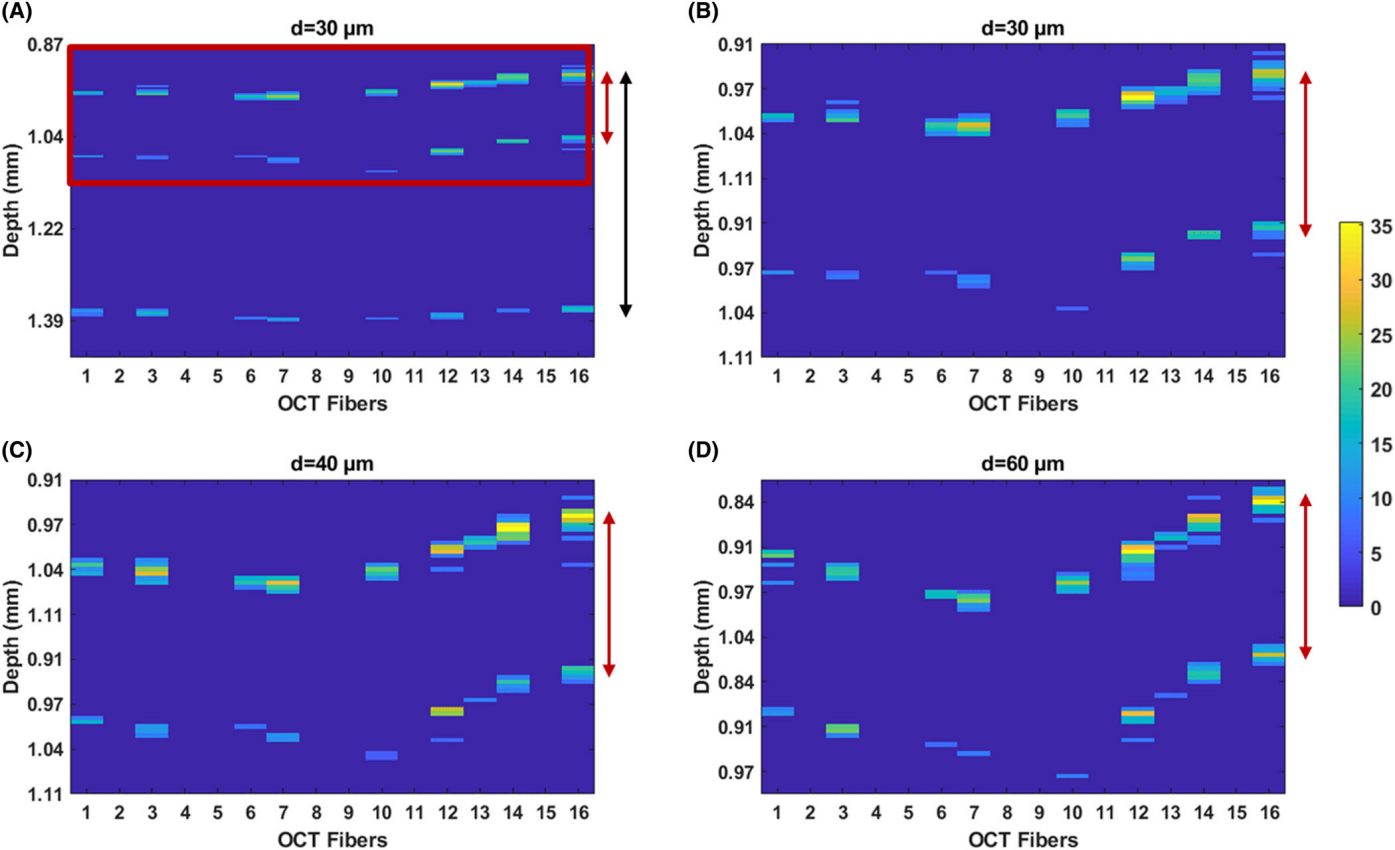
OCT B‐scan acquired during indentation at different depths on a thin PDMS sample. The red arrow indicates the front surface of the sample as detected from the top and bottom surface of the focusing element, whereas the black arrow in A indicates the thickness of the samples. *Y*‐axis depth: ∼600 µm (A) and ∼250 µm for (B), (C) and (D). The magnitude scale bar represents the A‐scan intensity in dB

To demonstrate the working principle of the probe, we performed a series of indentation experiments on a PDMS‐based phantom while recording the OCT signal collected by the OCT fibres. Figure 6 shows the result obtained at 3 different depths, namely, 30, 40 and 60 µm. The experiment demonstrates that the OCT data can reveal different deformation profiles under different indentation conditions. Specifically, in Figure 6(A), one can notice that three interfaces are detected: Peak 1 is the (fibre‐to‐) lens‐front material surface; peak 2 is the lens (‐to‐air)‐front material surface, whereas peak 3 represents the (fibre‐to‐) lens‐rear material interface. In order to appreciate micrometre deformation due to increasing the indentation depth, in Figures 6(B)‐(D), only the first surface of the PDMS sample (surface 1 and 2) is displayed for each indentation depth. The latter represents the reflection that originated from the focusing elements' top and bottom surface with the first PDMS layer. The distances measured for this set of data are ∼ *d_g_* = 65 µm (surface 1 vs surface 2 – red arrow in Figure 6A) and *d_g_* = 320 µm (surface 1 vs surface 2 – red arrow in Figure 6A). Similarly, the distance between the two surfaces detected in Figure 6(B)‐(D) by the sensor has been measured to be ∼*d_g_* = 65 µm for all the investigated indentation depth.

**FIGURE 7:**
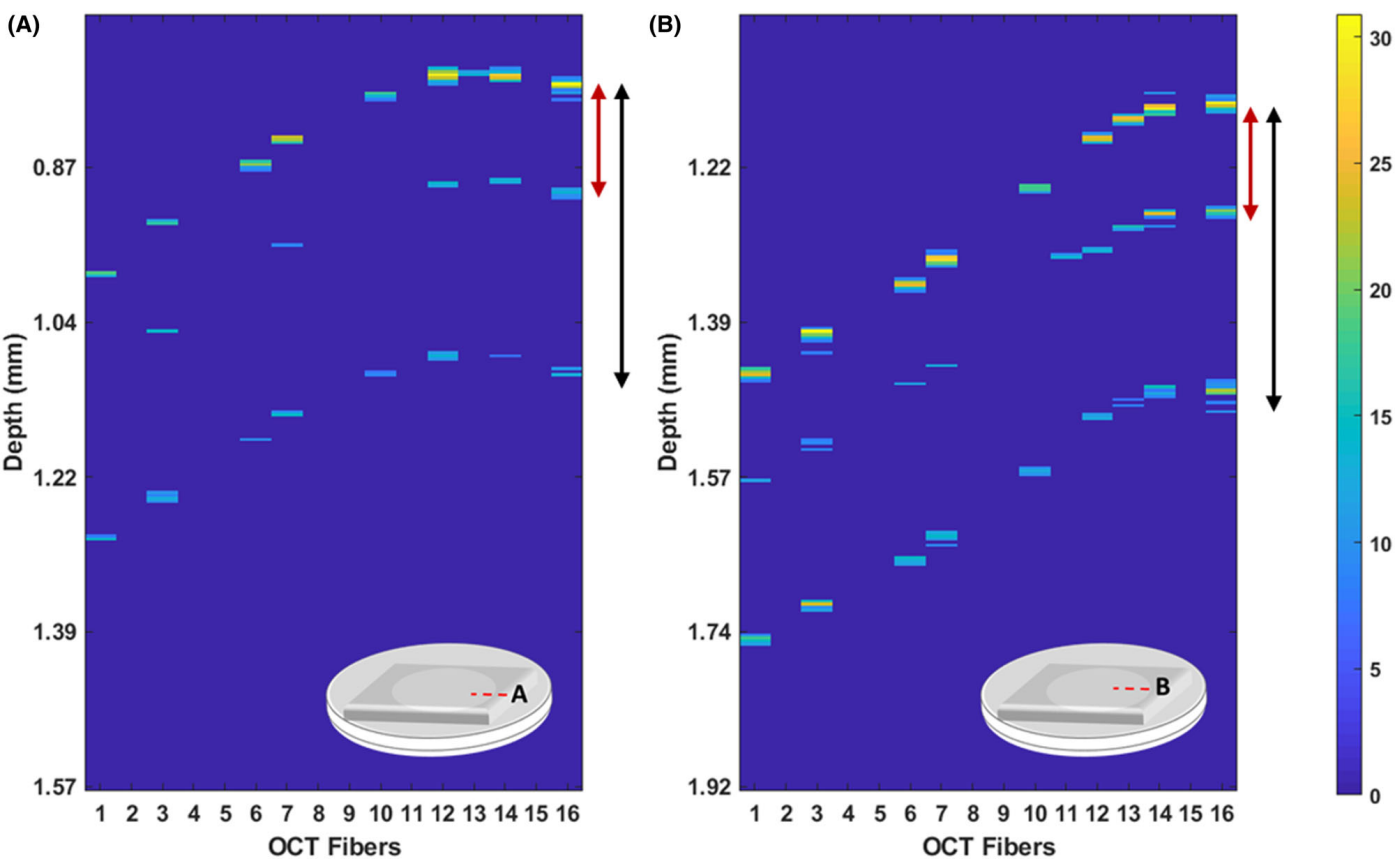
OCT B‐scan during indentation on thin PDMS sample: (A) the probe is position on the edge of the membrane; (B) the probe is moved far away from the edge. The red arrow indicates the front surface of the sample as detected from the top and bottom surface of the focusing element (peak 1 vs peak 2). The black arrow indicates the thickness of the PDMS samples (peak 1 vs peak 3). The magnitude scale bar represents the A‐scan intensity in dB

To further test the ability of the sensor to distinguish different types of deformations due to the indentation stroke, we performed two indentation experiments in two different locations of the PDMS‐based phantom. The first location was selected close to the rim of the ring over which the PDMS membrane is suspended (Figure 7A), while for the second indentation, the probe was positioned some 100 µm from the ring edge (Figure 7B). Our findings clearly show that both the indentation profiles are slightly asymmetrical since the probe is not positioned perfectly in the centre of the sample with respect to the ring. Moreover, the effect of the rigid substrate that lies around the membrane appears more evident in Figure 7(A) than Figure 7(B), as one would expect due to the indentation location. The *d_op_* between peak 1 and peak 2 (red arrow) is ∼126.8 µm as for Figures 5 and 6 and corresponds to the lens dimension (∼*d_g_* = 65 µm). On the contrary, the distance between peak 1 and 3 (black arrow) is, on average, ∼305 µm for both the figures and represents the PDMS membrane's thickness as measured with a commercial OCT system (see Figure S4).

## 4 DISCUSSION

Our work demonstrates that the general concept of a fibre‐array optomechanical sensor able to simultaneously indent and collect depth information of a phantom has the potential of assessing the morphological properties of materials while performing mechanical characterisation. Our findings prove that CP‐OCT systems based on lensed optical fibres can be successfully combined with a microindentation sensor.

However, it is fair to mention that the sensor still has several limitations. The assembling of the 16 OCT fibres on the 3D‐printed ferrule is tedious work, since the alignment, the mounting and the gluing is performed manually and under an optical microscope. Furthermore, the printing resolution of the grooves, created to precisely mount the OCT fibres, is smooth; therefore, the alignment in the *z*‐axis is suboptimal. Thus, more often than not, the OCT fibres are not pointing straight toward the sample, and the back coupling of the light from the sample back to the OCT fibres is usually weak, with significant deterioration of the OCT signal to the point that, for some fibres, the losses are so high that no A‐scan can be collected.

Although we recognise that several improvements in the fabrication steps need to be implemented, it is also important to highlight some strengths of the method. To the best of our knowledge, this is the first fibre array sensor able to collect depth profile and indentation simultaneously, without limitations on the sample thickness or preparation. The use of a high refractive index lens allows the imaging fibre array to be held far from the sample due to the depth‐of‐field offered by the barium titanate lenses. This feature leaves sufficient space for the cantilever to bend and probe the material without touching the OCT fibre lenses. Moreover, the integration of OCT imaging with indentation techniques could be further exploited by introducing ad hoc mathematical models able to extrapolate multiple information from single OCT depth profiles; one could think of providing a more extensive evaluation of deformation profiles in relation to the local material composition or to apply specific OCT algorithm for a more accurate extrapolation of peak profiles or other information hidden in the sample. Thus, the combination of OCT with micro indentation opens new ways to explore a whole range of information (eg sample topography, multilayered structures, thickness and mechanics) that are neglected while choosing either microindentation or optical imaging separately.

## FUNDING

This work has been financially supported by the European Research Council under the European Union's Seventh Framework Programme (FP/20072013)/ERC grant agreement no. [615170].

## DECLARATION OF INTEREST

D. Iannuzzi declares a potential conflict of interest as cofounder, shareholder and advisor of Optics11.

## Supporting information


**FIGURE S1** 3D‐printed ferrule‐top probe assembly: The 3D‐printed ferrule is mechanically embedded on a rigid arm used to mount the sensor on the *Z*‐piezoelectric actuator

**FIGURE S2** Sketches of the setup: A ferrule‐top probe is equipped with an optical fibre for interferometric readout of the cantilever and with a spherical tip to indent the sample. The 16‐OCT fibres are mounted on the top facet of the 3D printed ferrule (left). The sensor is then mounted on the *Z*‐piezoelectric actuator, which is solidly attached to an *XYZ* manipulator (right)

**FIGURE S3** Image of the setup: OCT imaging is obtained from the 16‐fibres connected to the Optical switch while the sensor hovers above the sample. Indentation measurements make use of the interferometric readout and are driven by the piezoelectric element

**FIGURE S4** PDMS membrane B‐scan: Commercial OCT B‐scan image of the PDMS sample employed for indentation. The thickness of the membrane is also shown

## References

[1] Weaver, V. M. (2017) Cell and tissue mechanics: The new cell biology frontier. Molecular Biology of the Cell, 28, 1815–1818. 28684606 10.1091/mbc.E17-05-0320 PMC5541832

[2] Fallenstein, G. T. , Hulce, V. D. , & Melvin, J. W. (1969) Dynamic mechanical properties of human brain tissue. Journal of Biomechanics, 2, 217–226. 16335085 10.1016/0021-9290(69)90079-7

[3] Efremov, Y. M. , Pukhlyakova, E. A. , Bagrov, D. V. , & Shaitan, K. V. (2011) Atomic force microscopy of living and fixed Xenopus laevis embryos. Micron (Oxford, England: 1993), 42, 840–852. 10.1016/j.micron.2011.05.010 21724405

[4] Bonilla, M. R. , Stokes, J. R. , Gidley, M. J. , & Yakubov, G. E. (2015) Interpreting atomic force microscopy nanoindentation of hierarchical biological materials using multi‐regime analysis. Soft Matter, 11, 1281–1292. 25569139 10.1039/c4sm02440k

[5] Dokukin, M. , & Sokolov, I. (2015) High‐resolution high‐speed dynamic mechanical spectroscopy of cells and other soft materials with the help of atomic force microscopy. Scientific Reports, 5, 1–14. 10.1038/srep12630 PMC4649865 26218346

[6] Cartagena, A. , & Raman, A. (2014) Local viscoelastic properties of live cells investigated using dynamic and quasi‐static atomic force microscopy methods. Biophysical Journal, 106, 1033–1043. 24606928 10.1016/j.bpj.2013.12.037 PMC4026794

[7] Thomas, G. , Burnham, N. A. , Camesano, T. A. , & Wen, Q. (2013) Measuring the mechanical properties of living cells using atomic force microscopy. Journal of visualized experiments: JoVE, 76, 1–8. 10.3791/50497 PMC3729185 23851674

[8] Beekmans, S. V. , & Iannuzzi, D . (2015) A metrological approach for the calibration of force transducers with interferometric readout. Surface Topography Metrology and Properties, 3, 025004

[9] Iannuzzi, D. , Heeck, K. , Slaman, M. , De Man, S. , Rector, J. H. , Schreuders, H. , … Deladi, S. (2007) Fibre‐top cantilevers: Design, fabrication and applications. Measurement Science & Technology, 18, 3247–3252.

[10] Chavan, D. , Van De Watering, T. C. , Gruca, G. , Rector, J. H. , Heeck, K. , Slaman, M. , & Iannuzzi, D . (2012) Ferrule‐top nanoindenter: An optomechanical fiber sensor for nanoindentation. Review of Scientific Instruments, 83(11), 1–6. 10.1063/1.4766959 23206101

[11] Van Hoorn, H. , Kurniawan, N. A. , Koenderink, G. H. , & Iannuzzi, D . (2016) Local dynamic mechanical analysis for heterogeneous soft matter using ferrule‐top indentation. Soft Matter, 12, 3066–3073. 26908197 10.1039/c6sm00300a PMC4819682

[12] Antonovaite, N. , Beekmans, S. V. , Hol, E. M. , Wadman, W. J. , & Iannuzzi, D . (2018) Regional variations in stiffness in live mouse brain tissue determined by depth‐controlled indentation mapping. Scientific Reports, 8, 12517. 30131608 10.1038/s41598-018-31035-y PMC6104037

[13] Marrese, M. , Antonovaite, N. , Nelemans, B. K. A. , Smit, T. H. , & Iannuzzi, D . (2019) Micro‐indentation and optical coherence tomography for the mechanical characterization of embryos: Experimental setup and measurements on chicken embryos. Acta Biomaterialia, 97, 524–534. 31377425 10.1016/j.actbio.2019.07.056

[14] Wintner, O. , Hirsch‐Attas, N. , Schlossberg, M. , Brofman, F. , Friedman, R. , Kupervaser, M. , … Buxboim, A. (2020) A unified linear viscoelastic model of the cell nucleus defines the mechanical contributions of lamins and chromatin. Advancement of Science, 7, 1901222. 10.1002/advs.201901222 PMC7175345 32328409

[15] Karoutas, A. , Szymanski, W. , Rausch, T. , Guhathakurta, S. , Rog‐Zielinska, E. A. , Peyronnet, R. , … Akhtar, A. (2019) The NSL complex maintains nuclear architecture stability via lamin A/C acetylation. Nature Cell Biology, 21, 1248–1260. 31576060 10.1038/s41556-019-0397-z

[16] Albers, P. T. M. , Govers, S. P. W. , Laven, J. , van der Ven, L. G. J. , van Benthem, R. A. T. M. , de With, G. , & Esteves, A. C. C. (2019) Design of dual hydrophobic–hydrophilic polymer networks for highly lubricious polyether‐urethane coatings. European Polymer Journal, 111, 82–94.

[17] Qazi, T. H. , Mooney, D. J. , Duda, G. N. , & Geissler, S. (2020) Niche‐mimicking interactions in peptide‐functionalized 3D hydrogels amplify mesenchymal stromal cell paracrine effects. Biomaterials, 230, 119639. 31776021 10.1016/j.biomaterials.2019.119639

[18] Yue, X. , Nguyen, T. D. , Zellmer, V. , Zhang, S. , & Zorlutuna, P. (2018) Stromal cell‐laden 3D hydrogel microwell arrays as tumor microenvironment model for studying stiffness dependent stromal cell‐cancer interactions. Biomaterials, 170, 37–48. 29653286 10.1016/j.biomaterials.2018.04.001

[19] Mattei, G. , Cacopardo, L. , & Ahluwalia, A. (2020) Engineering gels with time‐evolving viscoelasticity. Materials (Basel), 13, 438. 10.3390/ma13020438 PMC7014018 31963333

[20] Nguyen, D. T. , Nagarajan, N. , & Zorlutuna, P. (2018) Effect of substrate stiffness on mechanical coupling and force propagation at the infarct boundary. Biophysical Journal, 115, 1966–1980. 30473015 10.1016/j.bpj.2018.08.050 PMC6303235

[21] Moghaddam, A. O. , Wei, J. , Kim, J. , Dunn, A. C. , & Wagoner Johnson, A. J. (2020) An indentation‐based approach to determine the elastic constants of soft anisotropic tissues. Journal of the Mechanical Behavior of Biomedical Materials, 103, 103539. 31783285 10.1016/j.jmbbm.2019.103539

[22] Martorina, F. , Casale, C. , Urciuolo, F. , Netti, P. A. , & Imparato, G. (2017) In vitro activation of the neuro‐transduction mechanism in sensitive organotypic human skin model. Biomaterials, 113, 217–229. 27821307 10.1016/j.biomaterials.2016.10.051

[23] Arias, S. L. , Shetty, A. , Devorkin, J. , & Allain, J. P. (2018) Magnetic targeting of smooth muscle cells in vitro using a magnetic bacterial cellulose to improve cell retention in tissue‐engineering vascular grafts. Acta Biomaterialia, 77, 172–181. 30004023 10.1016/j.actbio.2018.07.013

[24] Xie, S. A. , Zhang, T. , Wang, J. , Zhao, F. , Zhang, Y. P. , Yao, W. J. , … Zhou, J. (2018) Matrix stiffness determines the phenotype of vascular smooth muscle cell in vitro and in vivo: Role of DNA methyltransferase 1. Biomaterials, 155, 203–216. 29182961 10.1016/j.biomaterials.2017.11.033

[25] Bokemeyer, A. , Tepasse, P. R. , Quill, L. , Lenz, P. , Rijcken, E. , Vieth, M. , … Bettenworth, D . (2019) Quantitative phase imaging using digital holographic microscopy reliably assesses morphology and reflects elastic properties of fibrotic intestinal tissue. Scientific Reports, 9, 1–11. 31852983 10.1038/s41598-019-56045-2 PMC6920451

[26] Chen, C. Y. , Caporizzo, M. A. , Bedi, K. , Vite, A. , Bogush, A. I. , Robison, P. , … Prosser, B. L. (2018) Suppression of detyrosinated microtubules improves cardiomyocyte function in human heart failure. Nature Medicine, 24, 1225–1233. 10.1038/s41591-018-0046-2 PMC6195768 29892068

[27] Amann, E. , Wolff, P. , Breel, E. , van Griensven, M. , & Balmayor, E. R. (2017) Hyaluronic acid facilitates chondrogenesis and matrix deposition of human adipose derived mesenchymal stem cells and human chondrocytes co‐cultures. Acta Biomaterialia, 52, 130–144. 28131943 10.1016/j.actbio.2017.01.064

[28] Chavan, D. , Mo, J. , de Groot, M. , Meijering, A. , de Boer, J. F. , & Iannuzzi, D . (2013) Collecting optical coherence elastography depth profiles with a micromachined cantilever probe. Optics Letters, 38, 1476. 23632523 10.1364/OL.38.001476

[29] Bartolini, L. , Feroldi, F. , Weda, J. J. A. , Slaman, M. , De Boer, J. F. , & Iannuzzi, D. (2017) Multimodal probe for optical coherence tomography epidetection and micron‐scale indentation, 10(6). 10.1142/S179354581742007X

[30] Gruca, G. , De Man, S. , Slaman, M. , Rector, J. H. , & Iannuzzi, D . (2010) Ferrule‐top micromachined devices: Design, fabrication, performance. Measurement Science & Technology, 21, 094033.

[31] Marrese, M. , Offerhaus, H. , Paardekam, E. , & Iannuzzi, D. (2018) 70 µm diameter optical probe for common‐path optical coherence tomography in air and liquids. Optics Letters, 43, 5929. 30547972 10.1364/OL.43.005929

[32] Marrese, M. , Lonardoni, D. , Boi, F. , van Hoorn, H. , Maccione, A. , Zordan, S. , … Berdondini, L. (2019) Investigating the effects of mechanical stimulation on retinal ganglion cell spontaneous spiking activity. Frontiers in Neuroscience, 13, 1–13. 31611765 10.3389/fnins.2019.01023 PMC6776634

[33] Beekmans, S. V. , & Iannuzzi, D . (2016) Characterizing tissue stiffness at the tip of a rigid needle using an opto‐mechanical force sensor. Biomedical Microdevices, 18, 1–8. 26838036 10.1007/s10544-016-0039-1 PMC4737792

[34] Bartolini, L. , Iannuzzi, D. , & Mattei, G. (2018) Comparison of frequency and strain‐rate domain mechanical characterization. Scientific Reports, 8, 1–11. 30209311 10.1038/s41598-018-31737-3 PMC6135832

