# Supplementary figures and images for: Indentation probe with optical fibre array‐based optical coherence tomography for material deformation
Source: J Microsc. 2021 Jan 13;282(3):205–14. doi: 10.1111/jmi.12994 (PMC8248032; doi:10.1111/jmi.12994)

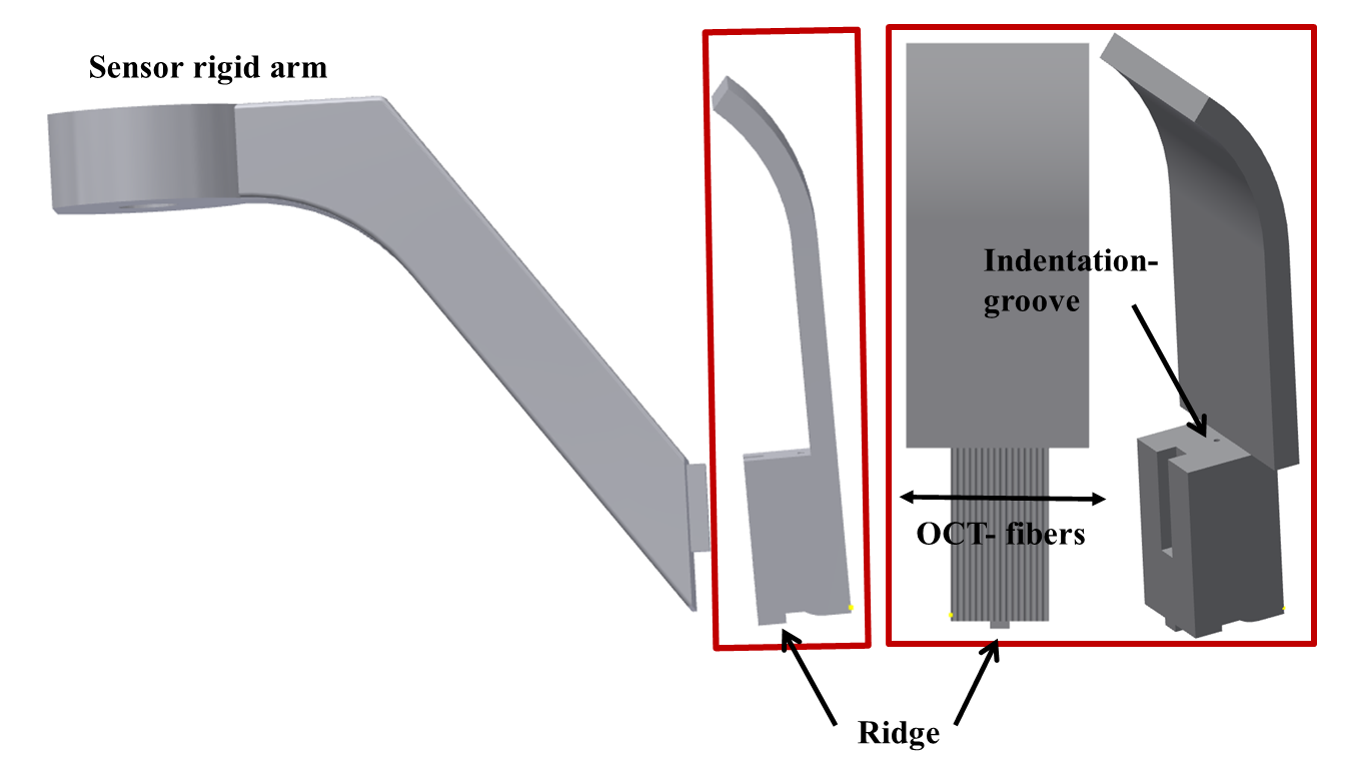

Supplement: Supplementary file 1 — FIGURE S1 3D‐printed ferrule‐top probe assembly: The 3D‐printed ferrule is mechanically embedded on a rigid arm used to mount the sensor on the Z‐piezoelectric actuator [file JMI-282-205-s004.tif]

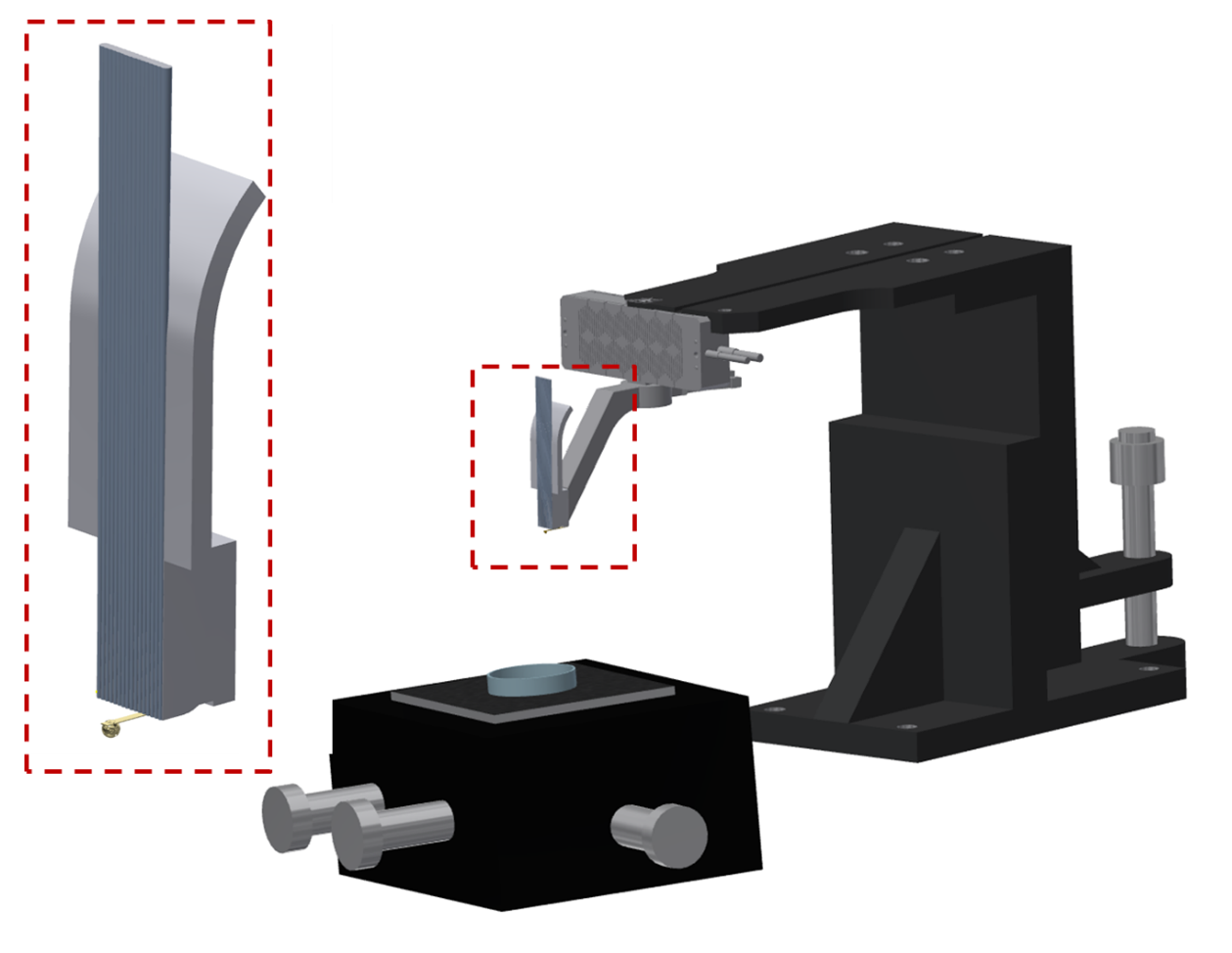

Supplement: Supplementary file 2 — FIGURE S2 Sketches of the setup: A ferrule‐top probe is equipped with an optical fibre for interferometric readout of the cantilever and with a spherical tip to indent the sample. The 16‐OCT fibres are mounted on the top facet of the 3D printed ferrule (left). The sensor is then mounted on the Z‐piezoelectric actuator, which is solidly attached to an XYZ manipulator (right) [file JMI-282-205-s002.tif]

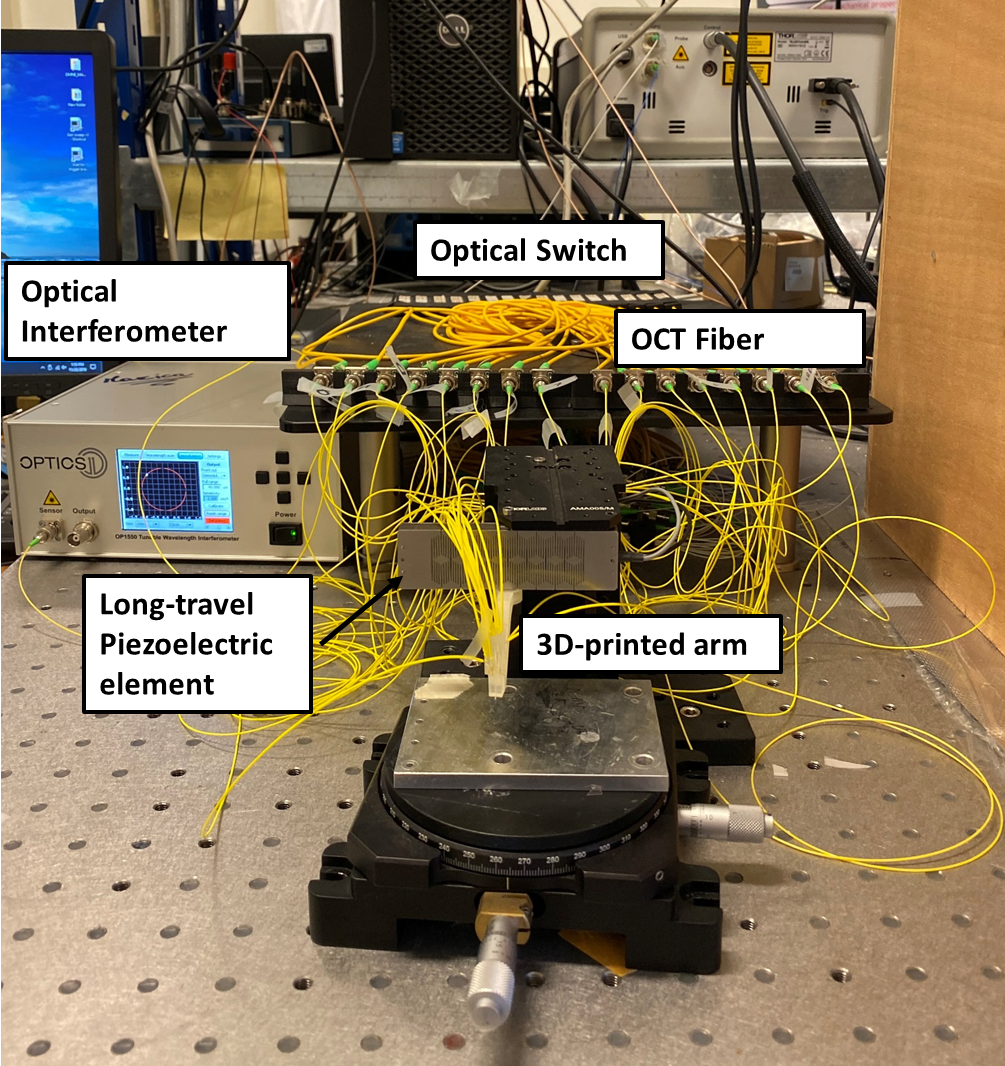

Supplement: Supplementary file 3 — FIGURE S3 Image of the setup: OCT imaging is obtained from the 16‐fibres connected to the Optical switch while the sensor hovers above the sample. Indentation measurements make use of the interferometric readout and are driven by the piezoelectric element [file JMI-282-205-s003.tif]

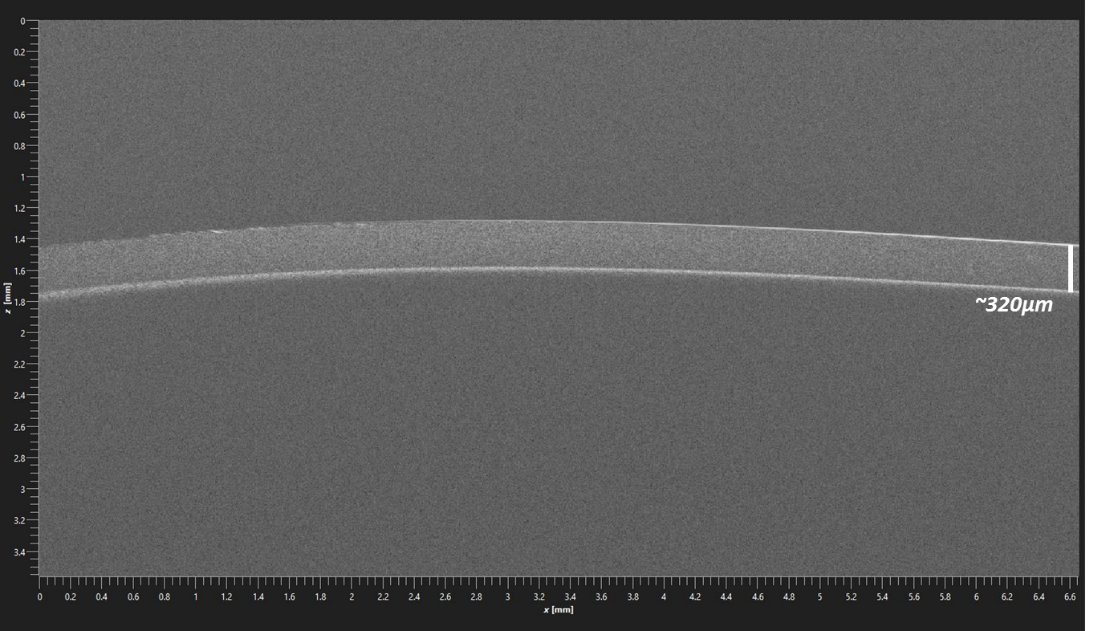

Supplement: Supplementary file 4 — FIGURE S4 PDMS membrane B‐scan: Commercial OCT B‐scan image of the PDMS sample employed for indentation. The thickness of the membrane is also shown [file JMI-282-205-s001.tif]
